# *Helicobacter pylori* co-infection with Epstein-Barr virus and the risk of developing gastric adenocarcinoma at an early age: Observational study infectious agents and cancer

**DOI:** 10.1016/j.amsu.2021.102651

**Published:** 2021-07-31

**Authors:** Fatima Ezzahra Rihane, Driss Erguibi, Othmane Elyamine, Berjas Abumsimir, Moulay Mustapha Ennaji, Farid Chehab

**Affiliations:** a Laboratory of Genetic and Molecular Pathology, Faculty of Medicine & Pharmacy Casablanca. University Hassan II of Casablanca., 20360, Morocco; b Laboratory of Virology, Microbiology, Quality, Biotechnologies/ Ecotoxicology and Biodiversity, Faculty of Sciences & Technologies Mohammedia. University Hassan II of Casablanca, 20650, Morocco; c Service of Digestive Cancers Surgery and Liver Transplant, Department of Surgery. Ibn Rochd University Hospital Center, Faculty of Medicine & Pharmacy Casablanca. University Hassan II of Casablanca, 20360, Morocco

**Keywords:** Gastric cancer, *Helicobacter pylori*, Epstein-barr virus, Co-infection, Age

## Abstract

**Background:**

Gastric cancer (GC) is one of the leading causes of morbidity and mortality worldwide. The onset and progression of gastric cancer are attributed to numerous triggers, these triggers may be infection of the gastric epithelium by *Helicobacter pylori* (*H. pylori*), or by Epstein-Barr virus (EBV). Both agents can establish a lifelong persistent infection in the host, leading to chronic inflammation, which also contributes to cancer development. Objective: The objective of this study is to present the status of co-infection with *H. pylori* and EBV and the risk of developing adenocarcinoma at an early age in the population of Grand Casablanca.

**Methods:**

In this study, 100 gastric tissue samples from patients with gastric cancer were examined for detection of *H. pylori* and EBV in tumor tissue using PCR techniques, and the clinical relevance was statistically analyzed.

**Results:**

Results revealed an individual Epstein-Barr virus (EBV) infection observed in (40 %) of gastric carcinoma cases. Furthermore, the frequency of EBV infection was significantly different with intestinal and diffuse gastric cancer types [15 % vs. 85 %; <0.05]. The prevalence of individual *H. pylori* infections was 34 %, while the frequency of co-infection was 16 %. Moreover, no significant association was found between co-infection and sex, tumor grade, stage, and lymph node metastasis, but there was a significant association between co-infection and the age of GC patients.

**Conclusion:**

Thus understanding the status of co-infection could clarify the process of gastric carcinogenesis, and application of this knowledge for clinical purposes could facilitate diagnosis, risk management, and prevention.

## Highlights

Determination of the frequency of *H. pylori* and EBV infections
Correlation between co-infection and age of gastric cancer patients
Evaluation of the clinicopathological features associated with the presence of infectious agents.

## 1. Introduction

Gastric cancer (GC), or stomach cancer, is the most prevalent malignancy in the world [1]. Despite the reduction in frequency and mortality rates in recent decades, it is still the fifth most common cancer and the third leading cause of cancer death worldwide, with An estimate of 1,033,701 new cases and 782,685 deaths related to GC recorded in 2018 [2].

GC is cancer with a recognized infectious etiology, involving viruses and bacteria. Recently, several studies have been carried out to understand the role of pathogens that infect the human stomach, especially *Helicobacter pylori* (*H. pylori*), which is considered the most common cause of gastric carcinogenesis, it is classified as a class 1 carcinogen by the World Health Organization [[3], [4], [5]]. And there is also the main risk factor that is the Epstein-Barr virus (EBV), involved in gastric carcinogenesis [6]. Both pathogens are usually acquired early in life, with about 50 % of the world's adult population infected with *H. pylori* and 90 % with EBV [7,8].

Carcinogenic pathogens are classified as acting directly or indirectly according to their mechanisms of transformation [9]. Although *H. pylori* infection is considered to stimulate a chronic inflammatory response leading to increased cell turnover that can result in the accumulation of mitotic errors, the virulence factor CagA (cytotoxin-associated gene), promotes directly mutations in cell cycle regulatory genes, deficiencies in DNA repair mechanisms, loss of cell adhesiveness and epigenetic changes that can alter cell function and promote cell autonomy and malignant transformation [10,11]. On the other hand, EBV is considered a direct transforming pathogen through the expression of its own death/proliferation regulatory genes, and it has been classified as a type I carcinogen by the International Agency for Research on Cancer (IARC) [[12], [13], [14]]. EBV infection has been associated with several types of B-cell lymphomas and upper gastrointestinal carcinomas, and it can infect the superficial epithelium of the stomach through B cells carrying reactivated EBV, which can trigger carcinogenesis [15,16]. Results from in vitro studies suggest that EBV-infected B cells generate a high level of infectivity on epithelial cells [17]. EBV can induce gastric epithelial cell death or persist as a latent infection and promote cancer progression [18]. Some gastric carcinomas harbor the EBV monoclonal genome in each cancer cell; this

Finding suggests that all these cells originated from the same infected progenitor cell, and the viral monoclonality in EBV-positive GC reinforces the causal relationship between EBV and gastric carcinogenesis [19,20].

The present study aimed to present the status of *H. pylori* co-infection with EBV and the risk of developing adenocarcinoma at an early age, as well as the evaluation of the clinicopathological features associated with the presence of infectious agents.

## 2 Materials and methods

### 2.1 Patients and samples

A total of 100 gastric tissue samples from patients who underwent gastric resection in the department of surgery of Ibn Rochd University Hospital Center, Casablanca, were included in this study. All clinical and pathological parameters were recorded by the physicians in the medical record registry the department of surgery of Ibn Rochd University Hospital Center, Casablanca.

The study was approved by the Biomedical Research Ethics Committee of the Faculty of Medicine and Pharmacy of Casablanca, Morocco (Reference number: No 13/19), and each subject signed informed consent.

### 2.2 DNA extraction for the detection of *Helicobacter pylori (H. pylori)* and Epstein-Barr virus (EBV)

Tissue samples were immediately frozen and stored at −80 °C until use. DNA was extracted from the tissues using the Pure link Invitrogen® Genomic DNA mini kit, Thermo Fisher USA, according to the manufacturer's instructions. The quality and quantity of the DNA obtained were evaluated using NanoDrop 2000 (Technologies, Wilmington, DE, USA).

### 2.3 Detection of *H. pylori* and EBV

*H. pylori* were detected in biopsies by PCR using glmM primers [21]. And the cagA status was checked using the primers as described previously [22]. EBV was detected using nested PCR, Primers for this virus was determined as previously described [23]. Briefly, PCR reaction was carried out in a 25 μl reaction mixture containing genomic DNA (8 ng), 2 × Taq PCR master mix kit Qiagen USA, 10 μmol forward and reverse primers. PCR amplification was performed using a PerkinElmer 2400 GeneAmp PCR System 2400 Thermal Cycler®, CA, USA. Using the primers indicated in Supplementary Table S1. Cycling conditions were as follows: denaturation at 94 °C for 3 min, followed by 35 cycles of denaturation at 94 °C for 1 min, annealing at the specific temperature for 1 min, extension at 72 °C for 1 min, and the reaction was finished with a 10 min extension at 72 °C. PCR products were size-fractionated by gel electrophoresis for 1.5 h at 70 V on 2 % agarose.

### 2.4 Statistical analysis

Statistical analysis was performed using SPSS 23.0 statistical software (SPSS, Inc., Chicago, IL, USA). The correlation between the different disease parameters was analyzed by the Student's t-test, the Chi-square test, and the Fisher exact test. The difference was considered significant when the p-value was less than 0.05.

The current paper has been formulated and reported following the STROCSS criteria [24].

## 3. Results

### 3.1. Clinicopathological characteristics

Table 1 represents the clinico-pathological characteristics of the recruited patients. Using the median age; which is 58 years (range, 36–72 years); all GC patients were divided into two age groups: patients aged 58 years or older and patients younger than 58 years.

**Table 1:** Patients’ clinico-pathological characteristics.

**Characteristics**	**%**
Age*	
<58 years	50
≥58 years	50
***Gender***	
Male	56
Female	44
***Lauren's classification***	
intestinal type	42
diffuse type	58
***Lymph node metastasis***	
Negative	28
Positive	72
***Stage***	
Low (I and II)	30
High (III and IV)	70

*The mean and median to 58, Therefore, we set this age as a dividing line between two age groups: <58 and ≥ 58.

Based on Lauren's classification, the most widely used system for gastric adenocarcinomas, there are two main types of gastric tumors: the diffuse type, which has a worse prognosis and is characterized by invasive growth and the absence of precancerous lesions, and the second one, which is intestinal type, whose development depends on environmental factors and is associated with precancerous lesions, especially chronic and atrophic gastritis, metaplasia and dysplasia [[25], [26], [27]]. According to this classification, 58 % of the samples were of the diffuse type and the remaining 42 % of the intestinal type. As shown in Table 1, according to the TNM classification [28], most samples were high stage with metastasis to lymph nodes.

### 3.2. *H. pylori* and EBV detection

Overall, *H. pylori* DNA was found in 34 % of GC samples, and the cagA was detected in all 34H. pylori-positive tissues. The EBV DNA was detected in 40 %, and the co-infection was detected in 16 % of GC samples.

### 3.3. Association between co-infection status and age

**Fig. 1:**
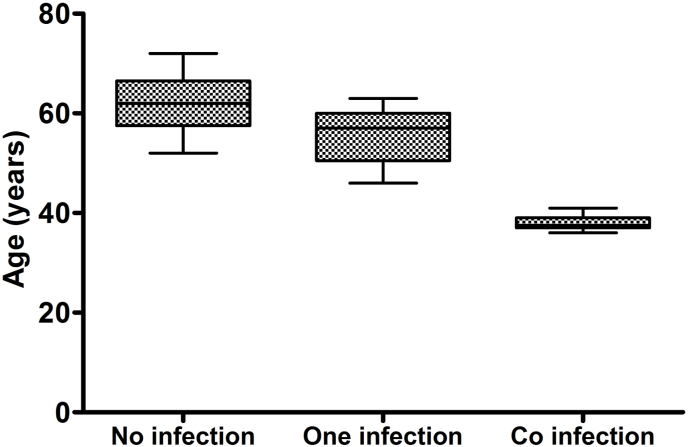
Association between the age of gastric cancer patients and their infection status.

A significant association between age and co-infection was observed. Fig. 1 shows that patients who had more than 1 infection were affected with GC at a significantly early age than those with no or 1 infection [*P*-value = 0.004].

### 3.4. Association between infections status and clinicopathological features

The association between the infection status of GC patients and their clinicopathological characteristics is presented in Table 2. Individual Epstein-Barr virus (EBV) infection was observed in (40 %) of GC cases. In addition, the frequency of EBV infection was significantly different between intestinal and diffuse gastric cancer [15 % versus 85 %; <0.05]. The prevalence of individual *H. pylori* infection was 34 % of GC samples, and we found that the presence of *H. pylori* in the diffuse type was higher than in the intestinal type. In addition, a significant association was found between *H. pylori* infection and differentiation, tumor stage, and lymph node metastasis. While the frequency of co-infection was 16 % of GC cases, and no significant association was found between co-infection and gender, stage, and lymph node metastasis.

**Table 2:** Association of coinfection with various clinico-pathological features of gastric cancer patients (n = 100).

Clinicopathological features	Cases	***H. pylori* status**		*P-value*	**EBV status**		*P-value*	Co-infection				*P-value*
		Negative	positive		negative	positive		negative		positive		
Age groupe*												
<58 years	50	24	26	0,007	26	24	0,25	34	16		0,004	
≥58 years	50	42	8		34	16		50	0			
***Gender***												
Male	56	36	20	0,77	34	22	0,91	44	12		0,44	
Female	44	30	14		26	18		40	4			
***Tumor size***												
<5 cm	20	20	0	0,01	18	2	0,03	20	0		0,18	
≥5	80	46	34		42	38		64	16			
***Histopathological differenciation***												
well	24	24	0	0,004	20	4	0,09	24	0		0,17	
Moderate/Poor	76	42	34		40	36		60	16			
***Lauren's classification***												
intestinal type	42	34	8	0,058	36	6	0,002	42	0		0,015	
diffuse type	58	32	26		24	34		42	16			
***Lymph node metastasis***												
Negative	28	26	2	0,01	24	4	0,026	28	0		0,087	
Positive	72	40	32		36	36		56	16			
***Lymphatic duct vessels invasion***												
Negative	30	28	2	0,008	24	6	0,059	30	0		0,86	
Positive	70	38	32		36	34		54	16			
***Stage***												
Low (I and II)	30	30	0	0,001	26	4	0,012	30	0		0,86	
High (III and IV)	70	36	34		34	36		54	16			

*The mean and median were equal to 58, Therefore, we set this age as a dividing line between two age groups: <58 and ≥ 58.

## 4 Discussion

Emerging evidence has demonstrated an association between cancers and infections by microorganisms, which may play a role in either the initiation of cancer cell growth or its maintenance [29,30]. While much of the gastrointestinal tract represents a favorable environment for microbial life, this is not the case for the stomach, where any microorganism would have to tolerate extremely acidic conditions, antimicrobial compounds, enzymes, and structural barriers [31]. Thus, to colonize the stomach, any pathogen must adapt to an extremely hostile and highly variable environment. In this regard, several studies have been conducted to understand the role of pathogens that infect the human stomach, particularly *H. pylori*, which is considered the most common cause of gastric carcinogenesis [[3], [4], [5]]. And there is also the main risk factor that is the Epstein-Barr virus (EBV), involved in gastric carcinogenesis [6]. Both pathogens are generally acquired early in life, with approximately 50 % of the world's adult population infected with *H. pylori* and 90 % with EBV [6,32].

The high prevalence of *H. pylori* in gastric tumors has been widely reported around the world [33,34]. A systematic review and meta-analysis showed that *H. pylori* eradication therapy was effective in reducing the incidence of GC [35]. In the present study, *H. pylori* were positive in 34 % of GC samples, and the cagA gene was detected in all *H. pylori-positive* tissues, confirming the involvement of the cagA gene in tumor progression. While the exact mechanism by which *H. pylori* can induce gastric carcinogenesis has not yet been fully elucidated, it is known that the inflammatory process induced by this bacterium, linked to genetic and epigenetic events in the host, is capable of inducing a cascade of morphological events, including precancerous and malignant transformations (intestinal or diffuse GC) [36,37]. In our study, we found that the presence of *H. pylori* in the diffuse type was higher than in the intestinal type, an association that was also confirmed in other studies conducted in locations with high incidence rates of GC [38,39]. In addition, a significant association was found between *H. pylori* infection and differentiation, tumor stage, and lymph node metastasis.

Regarding the involvement of EBV in the development of GC, this virus is usually present in about 10 % of GC cases [18,40]. Our study showed that the incidence of EBV infection in GC patients in our study population was 40 %, which is higher than that reported by previous studies in the United States and Germany (16%–26 %) [41,42]. Lower frequencies were found in Iran (3%–11 %) [43,44]. This discrepancy may be due to differences in environmental and geographic factors. In addition, the frequency of EBV infection was significantly different between intestinal and diffuse gastric cancer [15 % vs. 85 %; <0.05]. Some studies have shown a higher rate of EBV infection in men than in women [45,46], but our results did not show a significant association between EBV infection and gender.

The cooperation of infectious agents may have oncogenic influences or exacerbate their effect, relatively, several studies have shown that the presence of *H. pylori* promotes reactivation of the virus from its latent state in gastric epithelial cells, while Saiki et al. [47] proposed that the inflammatory stress generated by this bacterium may attract greater infiltration of EBV-carrying lymphocytes, increasing the possibility that epithelial cells will come into contact with these lymphocytes and thus be infected. EBV can also support *H. pylori*, Cárdenas-Mondragó et al. [48] found that EBV acts as a cofactor in triggering gastric inflammation with H. py*lori* in gastric diseases. In the present study, we sought to assess the prevalence of co-infections in GC tissues, and although the incidence of most cancers increases with age, our study indicated that early age of GC occurrence is significantly correlated with EBV *H. pylori* - cagA + co-infection. This suggests that co-infection promotes tissue malignancy, and the rate of EBV *H. pylori* - cagA + co-infection observed in patients with more aggressive tumors further supports the role of this interaction in the development and/or progression of gastric adenocarcinoma in the patients analyzed.

## 5 Conclusion

Our results indicate a high prevalence of infections in GC patients, but no significant association was found between infection and patient gender. However, there was a significant association between co-infection and the age of GC patients. Interestingly, infections have a positive feedback effect on cancer. Thus, information about the personal history of infection may help improve cancer prevention and treatment strategies. The observation of a high prevalence of infections in the present study suggests that a combination of traditional cancer therapies with antiviral and antimicrobial agents may offer a better chance of therapeutic success.

## Declaration of competing interest

The authors declare no conflict of interest.

## Author agreement statement

We the undersigned declare that this manuscript is original, has not been published before and is not currently being considered for publication elsewhere.

We confirm that the manuscript has been read and approved by all named authors and that there are no other persons who satisfied the criteria for authorship but are not listed. We further confirm that the order of authors listed in the manuscript has been approved by all of us.

We understand that the Corresponding Author is the sole contact for the Editorial process. He/she is responsible for communicating with the other authors about progress, submissions of revisions and final approval of proofs.
