# Supplementary material for: Helicobacter pylori co-infection with Epstein-Barr virus and the risk of developing gastric adenocarcinoma at an early age: Observational study infectious agents and cancer
Source: Ann Med Surg (Lond). 2021 Jul 31;68:102651. doi: 10.1016/j.amsu.2021.102651 (PMC8346356; doi:10.1016/j.amsu.2021.102651)
Supplement: Multimedia component 1 [file mmc1.docx]

**Table S1.** Primer Sequences and PCR conditions for detection of *Helicobacter pylori* (*H. pylori)* and Epstein-Barr virus (EBV)

| **Genes** | **Primer name** | **Sequence** | **Amplicon Length** | **T (°C)** | **Ref.** |
| --- | --- | --- | --- | --- | --- |
| glmM | P-F | AAGCTTTTAGGGGTGTTAGGGGTTT | 294 bp | 50 | [[21](#_ENREF_21)] |
|  | P-R | AAGCTTACTTTCTAACACTAACGC |  |  |  |
| cagA gene | P-F | AATACACCAACGCCTCCAAG | 400bp | 60 | [[22](#_ENREF_22)] |
|  | P-R | TTGTTGCCGCTTTTGCTCTC |  |  |  |
| EBNA-2 OP | P-F | GCGGGTGGAGGGAAAGG | not  applicable | 58 | [[23](#_ENREF_23)] |
|  | P-R | GTCAGCCAAGGGACGCG |  |  |  |
| EBNA-2 IP | P-F | AGGCTGCCCACCCTGAGGAT | 168 bp | 66 |  |
|  | P-R | GCCACCTGGCAGCCCTAAAG |  |  |  |
